# Supplementary material for: Pyruvate Dehydrogenase Kinase 1 inhibition mediated oxidative phosphorylation enhancement in cartilage promotes osteoarthritis progression
Source: BMC Musculoskelet Disord. 2023 Jul 20;24:597. doi: 10.1186/s12891-023-06585-6 (PMC10357736; doi:10.1186/s12891-023-06585-6)
Supplement: Supplementary file 1 — Additional file 1. [file 12891_2023_6585_MOESM1_ESM.zip › KEGG.Gsea.1653622432464/KEGG_TYROSINE_METABOLISM.html]

Details for gene set KEGG\_TYROSINE\_METABOLISM[GSEA]

|  || Dataset | 55457\_collapsed\_to\_symbols.cls.cls #OA\_versus\_normal.cls.cls #OA\_versus\_normal\_repos |
| Phenotype | cls.cls#OA\_versus\_normal\_repos |
| Upregulated in class | OA |
| GeneSet | KEGG\_TYROSINE\_METABOLISM |
| Enrichment Score (ES) | 0.4958204 |
| Normalized Enrichment Score (NES) | 1.9048501 |
| Nominal p-value | 0.0 |
| FDR q-value | 0.004820699 |
| FWER p-Value | 0.044 |
Table: GSEA Results Summary

  

Fig 1: Enrichment plot: KEGG\_TYROSINE\_METABOLISM      
 Profile of the Running ES Score & Positions of GeneSet Members on the Rank Ordered List

  

| SYMBOL | TITLE | RANK IN GENE LIST | RANK METRIC SCORE | RUNNING ES | CORE ENRICHMENT || 1 | GOT1 | glutamic-oxaloacetic transaminase 1 [Source:HGNC Symbol;Acc:HGNC:4432] | 59 | 0.974 | 0.0764 | Yes |
| 2 | BUD23 | BUD23 rRNA methyltransferase and ribosome maturation factor [Source:HGNC Symbol;Acc:HGNC:16405] | 210 | 0.813 | 0.1324 | Yes |
| 3 | COMT | catechol-O-methyltransferase [Source:HGNC Symbol;Acc:HGNC:2228] | 335 | 0.732 | 0.1836 | Yes |
| 4 | MIF | macrophage migration inhibitory factor [Source:HGNC Symbol;Acc:HGNC:7097] | 555 | 0.640 | 0.2198 | Yes |
| 5 | LCMT1 | leucine carboxyl methyltransferase 1 [Source:HGNC Symbol;Acc:HGNC:17557] | 735 | 0.585 | 0.2545 | Yes |
| 6 | HEMK1 | HemK methyltransferase family member 1 [Source:HGNC Symbol;Acc:HGNC:24923] | 742 | 0.583 | 0.3025 | Yes |
| 7 | LCMT2 | leucine carboxyl methyltransferase 2 [Source:HGNC Symbol;Acc:HGNC:17558] | 946 | 0.529 | 0.3307 | Yes |
| 8 | GSTZ1 | glutathione S-transferase zeta 1 [Source:HGNC Symbol;Acc:HGNC:4643] | 974 | 0.523 | 0.3722 | Yes |
| 9 | GOT2 | glutamic-oxaloacetic transaminase 2 [Source:HGNC Symbol;Acc:HGNC:4433] | 1308 | 0.457 | 0.3843 | Yes |
| 10 | TYR | tyrosinase [Source:HGNC Symbol;Acc:HGNC:12442] | 1547 | 0.421 | 0.4008 | Yes |
| 11 | HPD | 4-hydroxyphenylpyruvate dioxygenase [Source:HGNC Symbol;Acc:HGNC:5147] | 1681 | 0.402 | 0.4238 | Yes |
| 12 | TRMT11 | tRNA methyltransferase 11 homolog [Source:HGNC Symbol;Acc:HGNC:21080] | 1885 | 0.374 | 0.4392 | Yes |
| 13 | TAT | tyrosine aminotransferase [Source:HGNC Symbol;Acc:HGNC:11573] | 1979 | 0.363 | 0.4621 | Yes |
| 14 | MAOB | monoamine oxidase B [Source:HGNC Symbol;Acc:HGNC:6834] | 2192 | 0.339 | 0.4739 | Yes |
| 15 | FAH | fumarylacetoacetate hydrolase [Source:HGNC Symbol;Acc:HGNC:3579] | 2264 | 0.330 | 0.4958 | Yes |
| 16 | TYRP1 | tyrosinase related protein 1 [Source:HGNC Symbol;Acc:HGNC:12450] | 3555 | 0.198 | 0.4120 | No |
| 17 | ADH5 | "alcohol dehydrogenase 5 (class III), chi polypeptide [Source:HGNC Symbol;Acc:HGNC:253]" | 4720 | 0.094 | 0.3293 | No |
| 18 | ADH1C | "alcohol dehydrogenase 1C (class I), gamma polypeptide [Source:HGNC Symbol;Acc:HGNC:251]" | 4908 | 0.079 | 0.3213 | No |
| 19 | TH | tyrosine hydroxylase [Source:HGNC Symbol;Acc:HGNC:11782] | 5374 | 0.042 | 0.2887 | No |
| 20 | ALDH3B2 | aldehyde dehydrogenase 3 family member B2 [Source:HGNC Symbol;Acc:HGNC:411] | 5617 | 0.022 | 0.2718 | No |
| 21 | ALDH1A3 | aldehyde dehydrogenase 1 family member A3 [Source:HGNC Symbol;Acc:HGNC:409] | 5831 | 0.007 | 0.2557 | No |
| 22 | ALDH3B1 | aldehyde dehydrogenase 3 family member B1 [Source:HGNC Symbol;Acc:HGNC:410] | 5862 | 0.004 | 0.2538 | No |
| 23 | DBH | dopamine beta-hydroxylase [Source:HGNC Symbol;Acc:HGNC:2689] | 6133 | -0.015 | 0.2340 | No |
| 24 | AOX1 | aldehyde oxidase 1 [Source:HGNC Symbol;Acc:HGNC:553] | 6243 | -0.024 | 0.2275 | No |
| 25 | ALDH3A1 | aldehyde dehydrogenase 3 family member A1 [Source:HGNC Symbol;Acc:HGNC:405] | 6413 | -0.036 | 0.2174 | No |
| 26 | TPO | thyroid peroxidase [Source:HGNC Symbol;Acc:HGNC:12015] | 6467 | -0.040 | 0.2166 | No |
| 27 | METTL2B | "methyltransferase 2B, methylcytidine [Source:HGNC Symbol;Acc:HGNC:18272]" | 6522 | -0.044 | 0.2160 | No |
| 28 | MAOA | monoamine oxidase A [Source:HGNC Symbol;Acc:HGNC:6833] | 8271 | -0.177 | 0.0948 | No |
| 29 | NAA80 | "N-alpha-acetyltransferase 80, NatH catalytic subunit [Source:HGNC Symbol;Acc:HGNC:30252]" | 8328 | -0.180 | 0.1054 | No |
| 30 | DDC | dopa decarboxylase [Source:HGNC Symbol;Acc:HGNC:2719] | 8605 | -0.202 | 0.1007 | No |
| 31 | ADH7 | "alcohol dehydrogenase 7 (class IV), mu or sigma polypeptide [Source:HGNC Symbol;Acc:HGNC:256]" | 8906 | -0.225 | 0.0961 | No |
| 32 | ADH1B | "alcohol dehydrogenase 1B (class I), beta polypeptide [Source:HGNC Symbol;Acc:HGNC:250]" | 9036 | -0.235 | 0.1056 | No |
| 33 | ADH1A | "alcohol dehydrogenase 1A (class I), alpha polypeptide [Source:HGNC Symbol;Acc:HGNC:249]" | 9283 | -0.254 | 0.1076 | No |
| 34 | AOC3 | amine oxidase copper containing 3 [Source:HGNC Symbol;Acc:HGNC:550] | 9375 | -0.262 | 0.1223 | No |
| 35 | ADH6 | alcohol dehydrogenase 6 (class V) [Source:HGNC Symbol;Acc:HGNC:255] | 9504 | -0.274 | 0.1352 | No |
| 36 | HGD | "homogentisate 1,2-dioxygenase [Source:HGNC Symbol;Acc:HGNC:4892]" | 9669 | -0.289 | 0.1465 | No |
| 37 | PNMT | phenylethanolamine N-methyltransferase [Source:HGNC Symbol;Acc:HGNC:9160] | 9991 | -0.320 | 0.1481 | No |
| 38 | DCT | dopachrome tautomerase [Source:HGNC Symbol;Acc:HGNC:2709] | 10995 | -0.429 | 0.1058 | No |
| 39 | AOC2 | amine oxidase copper containing 2 [Source:HGNC Symbol;Acc:HGNC:549] | 11550 | -0.508 | 0.1050 | No |
Table: GSEA details [plain text format]

  

Fig 2: KEGG\_TYROSINE\_METABOLISM      
 Blue-Pink O' Gram in the Space of the Analyzed GeneSet

  

Fig 3: KEGG\_TYROSINE\_METABOLISM: Random ES distribution      
 Gene set null distribution of ES for **KEGG\_TYROSINE\_METABOLISM**
